# Development and validation of a telephone classification interview for common chronic headache disorders

**DOI:** 10.1186/s10194-018-0954-z

**Published:** 2019-01-08

**Authors:** Rachel Potter, Siew Wan Hee, Frances Griffiths, Katherine Dodd, Eleanor Hoverd, Martin Underwood, Manjit Matharu

**Affiliations:** 1 Warwick Clinical Trials Unit, Warwick Medical School, University of Warwick, Coventry, CV4 7AL UK; 2 Division of Health Sciences, Warwick Medical School, University of Warwick, Coventry, CV4 7AL UK; 3 Warwick Medical School, University of Warwick, Coventry, CV4 7AL UK; 4 Headache Group, UCL Institute of Neurology and The National Hospital for Neurology and Neurosurgery, Queen Square, London, WC1N 3BG UK

**Keywords:** Chronic headache, Classification, Telephone interviews, Development, Validation, Primary care

## Abstract

**Background:**

For a trial of supportive self-management for people with chronic headache we needed to develop and validate a telephone classification interview that can be used by a non-headache specialist to classify common chronic headache types in primary care. We aimed to specifically: exclude secondary headaches other than medication overuse, exclude primary headache disorders other than migraine and tension type headache (TTH), distinguish between chronic migraine and chronic TTH, and identify medication overuse headache.

**Methods:**

We held a headache classification consensus conference to draw on evidence and expertise to inform the content of a logic model underpinning the classification interview. Nurses trained to use the logic model did telephone classification interviews with participants recruited from primary care. Doctors specialising in headache did a second validation interview.

**Results:**

Twenty-six delegates attended the headache classification conference including headache specialist doctors, nurses and lay representatives (with chronic headache). We trained six nurses to do the classification interviews and completed 107 paired interviews, median days between interviews was 32 days (interquartile range 21–48 days). We measured level of agreement between the nurse and doctor interviews using proportion of concordance, simple kappa and prevalence-adjusted bias-adjusted kappa (PABAK). Proportion of concordance of agreement between nurse and doctor interviews was 0.76, simple kappa coefficient κ 0.31 (95% CI, 0.09 to 0.52), and PABAK 0.51 (95% CI, 0.35 to 0.68), a moderate agreement. In a sensitivity test following review of headache characteristics recorded, concordance was 0.91, κ = 0.53 (95% CI, 0.28 to 0.79), and PABAK = 0.81 (95% CI, 0.70 to 0.92), a very good agreement.

**Conclusion:**

We developed and validated a new evidence-based telephone classification interview that can be used by a non-headache specialist to classify common chronic headache types in primary care.

**Electronic supplementary material:**

The online version of this article (10.1186/s10194-018-0954-z) contains supplementary material, which is available to authorized users.

## Background

Despite established diagnostic criteria for different headache types [1, 2] the diagnosis of chronic headache can be a challenge for the non-expert clinician [3]. Many people with chronic headache disorders do not have an accurate diagnosis and receive inappropriate treatment of their headaches; in particular, there is under recognition of medication overuse headache [4].

The Chronic Headache Education and Self-management Study (CHESS) is a National Institute for Health Research (NIHR) funded programme grant (RP-PG-1212-20,018) with the overall aim of developing and evaluating an education and self-management programme for people living with chronic headache. As part of the study we needed to develop a telephone interview that can be used by a non-headache specialist nurse to confirm study eligibility and classify common chronic headache types in participants identified from primary care. Telephone interviews have been found to be as effective as face-to-face interviews in the diagnosis of migraine [5]. Additionally, for those allocated to the active intervention we needed an approach to inform the content of a one-to-one nurse interview that follows on from the group education and self-management intervention. Specifically we needed to be able to:

- Confirm that participants have headache on ≥15 days per month for ≥3 months
- Exclude serious pathology (secondary headaches other than medication overuse headache)
- Exclude primary headache disorders other than migraine and tension type headache
- Distinguish between chronic migraine, probable chronic migraine, and chronic tension type headache (TTH)
- Identify medication overuse headache (MOH)

There is a distinction between diagnostic criteria primarily used in clinical care and classification criteria primarily used to define cohorts for research purposes [6]. Here we describe the development and validation of a telephone headache classification interview which allows us to both describe our study population and to use as part of our study intervention to provide participants with evidence-based advice based on their headache classification. The classification interview is not intended as a substitute for a clinical diagnosis [7].

We first did a systematic literature review to identify any existing tools used to classify or diagnose different headache types [8].We identified 30 tools, nine for multiple headache types and 21 for one headache type only, but none validated in primary care that can be used by a non-headache specialist to classify common headache disorders and screen for primary headaches other than migraine and TTH. As the review did not identify any tools that could be used for the CHESS study we needed to develop our own.

The aim of this study was therefore to develop and validate a telephone classification interview that can be used by a non-headache specialist to classify common chronic headache disorders in primary care.

## Methods

### Development of the classification interview

**Fig. 1:**
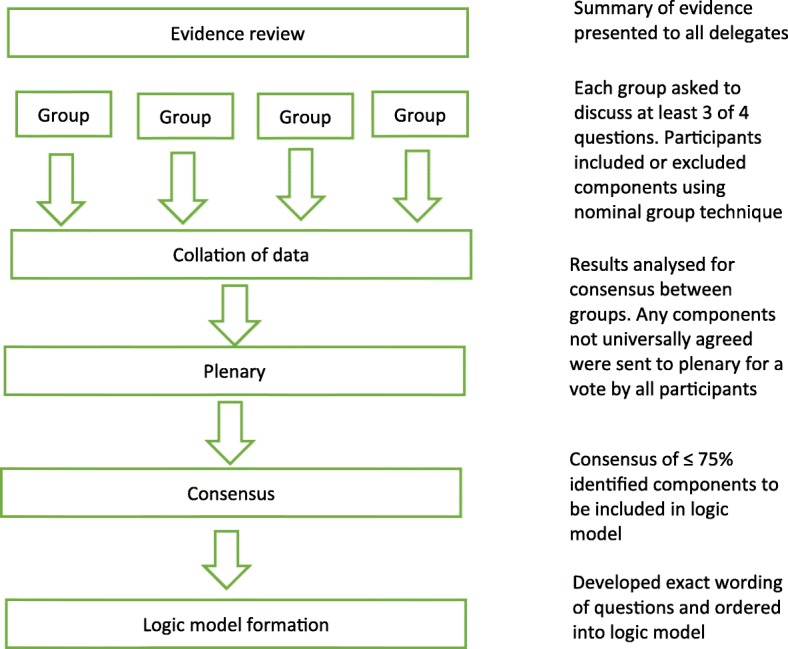
Overview of the consensus process

Our starting point were the findings from our systematic review [8]. This provided us with a summary of what was known about headache classification tools which we presented to participants at a headache classification consensus conference at the University of Warwick in October 2015. The aim of the meeting was to draw on evidence and expertise to reach consensus on questions to inform the content of a telephone headache classification interview that can be used by a non-headache specialist. Figure 1 provides an overview of the consensus process.

We invited headache specialist neurologists, headache specialist nurses, lay representatives (people with chronic headaches) and General Practitioners (GPs) with a specialist interest in headache identified through professional contacts, professional specialist interest groups and headache charity groups across the UK. We used a nominal group technique [9] to allow generation of ideas across disciplines and ensure consensus on key issues important to both health professionals and people living with chronic headache.

We briefed delegates on existing classification tools, key definitions, the aims and purpose of the classification interview, and randomly allocated within professional groups to one of four groups. Each group had a facilitator and a scribe who had been trained for the consensus process and an expert (researchers from the study team). Experts, from the study team, were not active in group discussions unless prompted to clarify specific points. Groups were asked to discuss four key questions, with each group starting on a different question and progressing through the questions in a pre-allocated sequence, and encouraged to complete at least three of the four questions:

- What do we need to know from a person to **exclude secondary headaches**?
- What do we need to know from a person to **exclude primary headaches other than chronic migraine and tension type headache**?
- What do we need to know from a person to **distinguish between chronic tension-type headache and chronic migraine**?
- What do we need to know from a person to **identify medication overuse headache**?

With guidance from facilitators, delegates were encouraged to identify and agree on the key items needed to address each question. Where there was uncertainty within the group about an item, the item was taken forward to a plenary session along with items that were not consistent across all four groups. At the plenary, delegates agreed on 75% as an acceptable level of consensus, and voted to include or exclude items before ranking them in order of importance. Following discussion and voting, consensus was achieved on the essential components of the telephone classification interview.

The following day, a small team of researchers met to interpret the results of the consensus conference and create mind maps to represent the finding for each of the key questions. These were used to inform the content and flow of a logic model which starts with questions to be asked of all interviewees then depending on the answers takes the interviewer down different question pathways. This logic model underpins the headache classification interview. Although the classification interview is based around the logic model, it is not intended to be a rigid interview schedule. Instead, the non-headache specialist doing the interview is encouraged to use the logic model to inform their clinical reasoning and decision-making. The structure and sequence of the telephone interview will be determined by individual consultation style, questioning, and by the participant’s responses.

### Validation of the classification interview

To validate the classification interview we did paired telephone interviews with participants recruited from primary care as part of a feasibility study for CHESS. The feasibility study received Ethics approval from West Midlands – Black Country Research Ethics Committee (15/WM/0165).

We identified participants from searches of general practice records from 14 practices in the West Midlands region of the UK that cover urban, small town and semi-rural areas with varying levels of deprivation and ethnic diversity. We searched for people who had consulted within the previous year with headache, or had been prescribed migraine specific medication. Due to the typically imprecise coding of chronic headache in primary care and the fluctuating nature of headache frequency the search included people with both episodic and chronic headaches. GPs screened lists of participants identified from the searches and excluded those with known serious underlying pathology or secondary causes of headache (other than medication overuse headache) or terminal illness because we did not want to cause unnecessary upset or distress inviting them to take part in a study of a self-management intervention for chronic headache. People interested in the study were contacted by a member of the study team to confirm that they had experienced headaches for at least half the days of the month and for at least three months. People who met these criteria and provided written consent were invited to take part in two telephone interviews, the first a telephone classification interview conducted by specially trained nurses and the second a validation interview conducted by doctors working for the National Migraine Centre. The nurse interviews were audio recorded for quality assurance purposes.

### Sample size and statistical analysis

We wanted to measure how well two raters (doctors and nurses) agreed on the classification of definite chronic migraine, probable chronic migraine and chronic TTH and presence of MOH as a nominal scale. Cohen’s kappa is the most commonly used summary to describe agreement. We made two comparisons; chronic migraine (definite or probable) versus chronic TTH, and with MOH versus without MOH. Assuming the level of agreement under the null hypothesis is 0.6 against the alternative of 0.8 (a substantial agreement [10] then at a two-sided alpha of 0.025 (correcting for multiplicity), power of 80% and the probability that a rating is a success is 0.5 then the required sample size is 153 [11]. To account for both prevalence and bias, a prevalence-adjusted bias-adjusted kappa (PABAK) was estimated in addition to the kappa statistic. We computed the confidence intervals for the estimated kappa and PABAK using Donner’s goodness of fit method [11]. We summarised demographics as mean and standard deviation or frequency and percentage as appropriate.

### Training to use the classification interview

We invited six nurses to attend a one day training workshop; all were research nurses and non-headache specialists with at least 10 years of nursing experience (range 10–35 years). The training was led by a consultant neurologist specialised in headache (MM), and included: headache assessment, history taking, secondary headache disorders, red flags in headache, primary headache disorders and classification of chronic headache types using the logic model. The nurses also received advice on telephone consultation techniques, and had the opportunity to observe and discuss example case scenarios.

Following the training workshop each nurse met one-to-one with a member of the study team for between one and two hours to ensure they felt confident to use the logic model, and practised classification interviews using mock scenarios. The nurse training was supplemented by a training manual providing a step by step guide on how to use the logic model to inform decision making. We also provided a telephone classification interview guide intended to be used during the classification interview with questions and prompts to guide the flow of the interview and allow the nurse to record participants’ responses.

After all the classification interviews had been completed we asked the nurses to complete a short anonymous online survey asking about their experience of the training and conducting the interviews. The doctors from the National Migraine Centre were asked to use their usual approach to a telephone assessment of headache type to complete the validation interviews and were not provided with a copy of the logic model.

## Results

### Development of the classification interview

Twenty six delegates attended the headache classification conference: five headache specialist nurses, 13 neurologists (10 with a specialist interest in headache), seven lay representatives (with chronic headache) and one GP with a specialist interest in headache.

Results from the plenary session for each of the four key questions are presented in Additional file 1.

#### What do we need to know from a person to exclude secondary headaches?

The four groups had very different approaches to how they addressed this question reporting between one and nine items to consider. All groups considered the length of time a person has a headache to be an essential item, with the assumption that a long duration rules out any serious pathology. Ruling out neurological signs and cerebrospinal fluid (CSF) pressure-related symptoms was also found to be important to the delegates and these items were voted to be kept in at the plenary session.

#### What do we need to know from a person to exclude primary headaches other than chronic migraine and tension type headache?

This question promoted a lot of discussion and all groups returned substantial responses. One group maintained an open question style, whereas the other groups decided that closed, symptom specific questions, were more appropriate to rule out primary headaches. At the plenary session, consensus was reached to keep in headache duration, location, autonomic features, presence or absence of restlessness and the timing of a headache attack.

#### What do we need to know from a person to distinguish between chronic tension-type headache and chronic migraine?

The group discussion centred on defining the key features of chronic migraine and chronic tension type headache. At plenary, headache characteristics, associated symptoms, and loss of function were considered to be the most pertinent points for a telephone classification system. The inclusion of a question relating to aura received 74% consensus at the plenary vote, and a decision was made by the study team to include this item.

#### What do we need to know from a person to identify medication overuse headache?

This question was not discussed by one group due to time restraints. For the remaining groups, most items related to the recognised definition of medication overuse headache, ‘headache occurring on 15 or more days per month taking acute or symptomatic headache medication (on 10/15 or more days per month, depending on the medication) for more than 3 months’.

Additional items focused on the other comorbidities that would cause patients to take pain relief medication, what happens when medication is withdrawn, and reliance on medication for daily function. Plenary voting for this question was the most decisive of the four questions.

### Development of the classification interview logic model

**Fig. 2:**
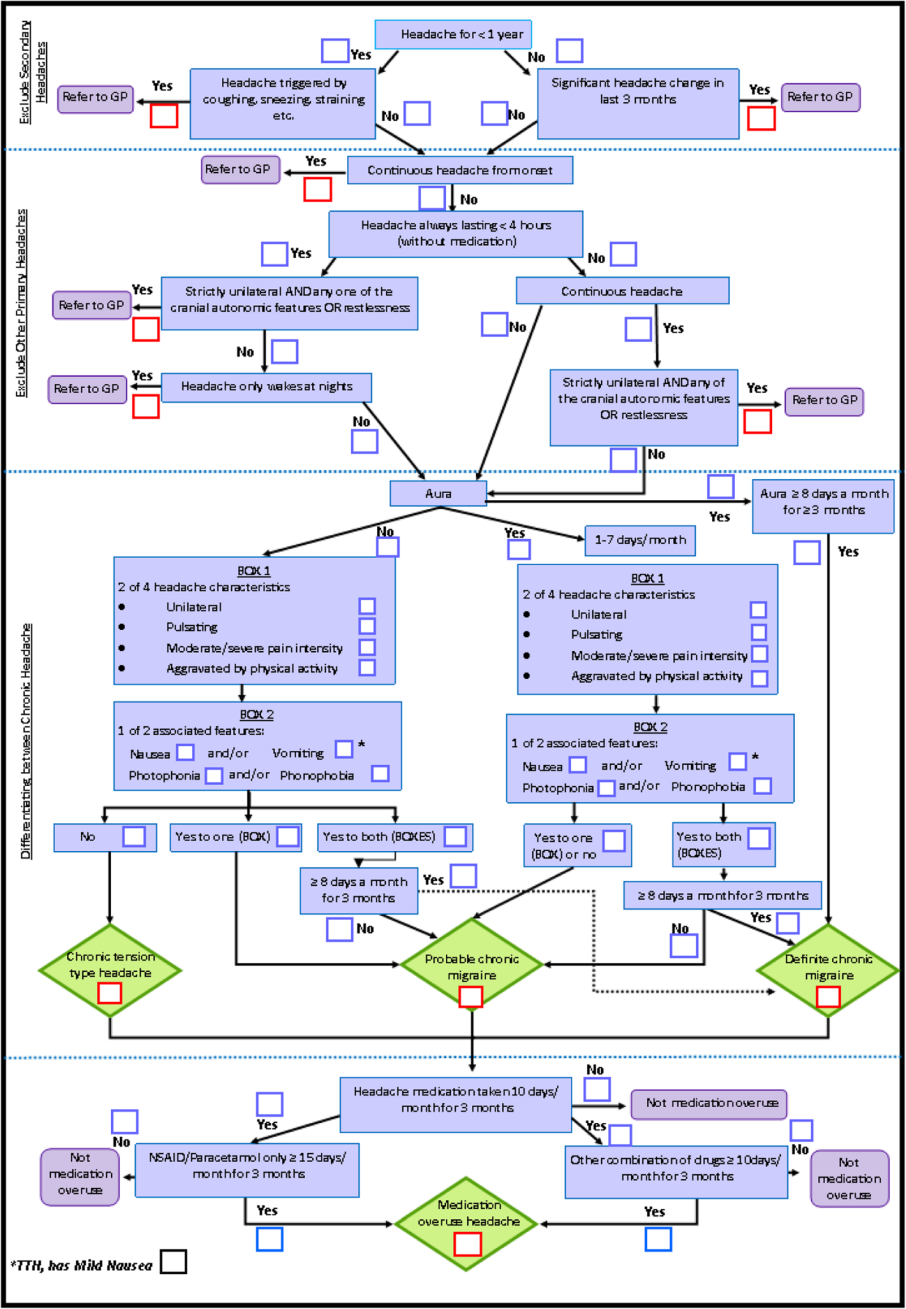
Classification interview logic model

The study team used the output from the consensus conference to develop a logic model that incorporated the findings from the day (Fig. 2). This included explicitly addressing questions to exclude other primary headaches and secondary headaches. Where there was lack of detail from the output from the consensus meeting the study team drew on their experience and standard definition of headache types to ensure there was clarity for the nurse doing the interviews. The team developed a five step model that clarifies that chronic headaches is present, excludes people with symptoms suggestive of secondary causes of chronic headache and primary headache disorders other than migraine or chronic tension type headache. Those with symptoms suggestive of these headache types are then out of the model and no further steps taken to clarify the classification within the interview. Any further diagnostic assessment would be carried out by their usual medical advisor. These three steps define the population of interest for the CHESS trial. The next step classifies population as chronic tension type, probable chronic migraine, or definite migraine in line with ICHD-IIIβ criteria [2]. Finally, the presence or absence of medication overuse is assessed.

### Validation of the classification interview

**Fig. 3:**
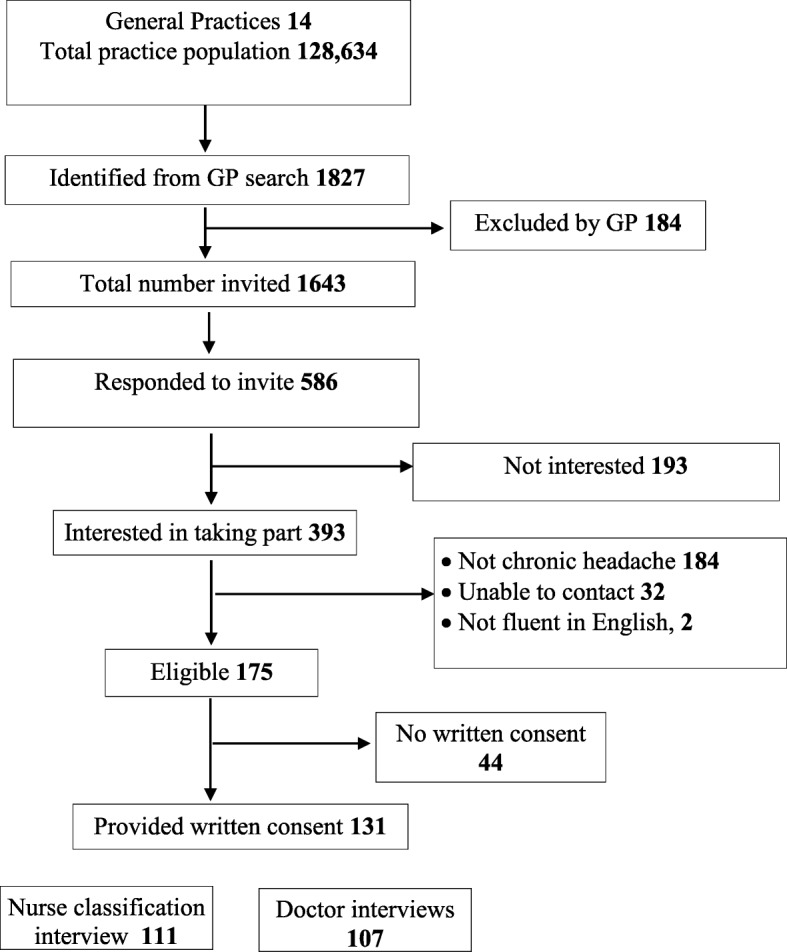
Flow diagram of practice and participant recruitment

We wrote to 1634 people identified from electronic database searches in the 14 general practices, 586 (36%) replied and of those that replied 393 (67%) expressed interest in the study; of these, an eligibility telephone call confirmed that 174 had headache ≥15 days/month for ≥3 months. We obtained written consent to take part in the validation study from 131 participants; six later withdrew from the study (Fig. 3). The mean age was 49 years (standard deviation, SD, 13), 108 (84%) females, 121 (93%) of White ethnicity and 86 (68%) were employed.

Nurses completed headache classification interviews with 111 participants and classified 46 (42%) with definite chronic migraine, 45 (41%) with probable chronic migraine and 4 (4%) with chronic TTH. Five (5%) participants were classified with other chronic headache types and 11 (10%) with non-chronic headache. Of the 95 participants classified with either chronic migraine (definite or probable) or chronic TTH, 63 (66%) had MOH; 97% chronic migraine and MOH and 3% chronic TTH and MOH.

Doctors from the National Migraine Centre completed diagnostic interviews with 108 participants and classified 52 (48%) with definite chronic migraine, 28 (26%) with probable chronic migraine and 4 (4%) with chronic TTH. One (1%) participant was classified with other chronic headache type and 23 (21%) were classified with non-chronic headache. Of the 84 participants classified with either chronic migraine or chronic TTH, 59 (70%) also had MOH (all with chronic migraine and MOH).

We did 107 paired classification interviews (Table 1). The median time between nurse and doctor interviews was 32 days (interquartile range, IQR, 21–48 days).

**Table 1:** Frequency of agreement and disagreement between nurse and doctor classification interviews

		Nurses’ classification					
		Definite chronic migraine	Probable chronic migraine	Chronic tension type headache	Other chronic headache	Non-chronic headache	Total
Doctors’ classification	Definite chronic migraine	28	18	1	3	1	51
	Probable chronic migraine	11	13	0	2	2	28
	Chronic tension type headache	0	2	1	0	1	4
	Other chronic headache	0	0	1	0	0	1
	Non-chronic headache	7	8	1	0	7	23
	Total	46	41	4	5	11	107

In the first agreement test, data from definite and probable chronic migraine were grouped as chronic migraine whereas chronic TTH, other chronic headaches and non-chronic headaches were grouped as ‘others’. We grouped definite and probable chronic migraine as chronic migraine for this analysis because this is the most useful classification for the purposes of informing treatment choices.

The proportion of concordance shown in Table 2 was 0.76, a good agreement between nurse and doctor interviews. However, the simple kappa coefficient, κ, was 0.31 (95% confidence interval, CI, 0.09 to 0.52), a fair agreement. The maximum attainable kappa, κ_max_, was 0.79, a good agreement, which reflects the strength of agreement while preserving the distribution of the marginal totals. Adjusting for prevalence and the distribution of the marginal totals, the prevalence-adjusted bias-adjusted kappa (PABAK) was 0.51 (95% CI, 0.35 to 0.68), a moderate agreement.

**Table 2:** Summary of proportion of concordance, simple kappa coefficient, κ, (95% confidence interval, CI), maximum attainable kappa, κ_max_, and prevalence-adjusted bias-adjusted kappa, PABAK (95% CI)

Comparison	Proportion of concordance	κ	95% CI	κ_max_	PABAK	95% CI
Chronic migraine vs. others^a^	0.76	0.31	(0.09 to 0.52)	0.79	0.51	(0.35 to 0.68)
Chronic migraine vs. chronic TTH^b^	0.96	0.38	(−0.18 to 0.94)	0.79	0.92	(0.83 to 1.00)
With MOH vs. without MOH^c^	0.76	0.40	(0.17 to 0.63)	0.93	0.51	(0.32 to 0.71)

^a^Chronic migraine = definite chronic migraine or probable chronic migraine; Others = chronic tension type, other chronic headaches or non-chronic headaches

^b^Chronic migraine = definite chronic migraine or probable chronic migraine; chronic TTH = chronic tension type headache

^c^MOH, medication overuse headache

In a sensitivity agreement test between chronic migraine and chronic TTH the agreement between nurses and doctors were better with κ = 0.38 (95% CI, 0.22 to 0.54), κ_max_ = 0.56, and PABAK = 0.76 (95% CI, 0.61 to 0.91), a good agreement. Of the paired 74 classifications of either chronic migraine or chronic TTH both nurse interviews and doctor interviews agreed that 12 (16%) did not have MOH and that 44 (59%) had MOH (see Table 3). The agreement between nurses and doctors generally moderately good; κ = 0.43 (95% CI, 0.26 to 0.61), κ_max_ = 0.79, and PABAK = 0.41 (95% CI, 0.20 to 0.61).

**Table 3:** Frequency of agreement and disagreement between nurse and doctor classification interviews for medication overuse headache

		Nurses’ classification		
		No	Yes	Total
Doctors’ classification	No	12	10	22
	Yes	8	44	52
	Total	20	54	74

We reviewed cases where both parties disagreed on the classification and when both classified the headaches as ‘others’ (non-chronic migraine or non-chronic TTH). There was disagreement on 29 headache classifications and seven classified as ‘others’. In most cases the disagreement was because the doctor had classified the headache type as episodic migraine and the nurse chronic migraine. Following the review of the headache characteristics recorded in the case report forms, 22 participants would have been classified as either having chronic migraine or chronic TTH, and three would have been excluded by both nurses and doctors. Of the three who would have been excluded, two had cluster headache and one had hemicrania continua. However, one participant would have been erroneously identified as having chronic TTH when in fact they had primary stabbing (ice pick) headache. Table 4 shows the frequency of agreement and disagreement from reclassifying these cases based on doctors’ and nurses’ notes. In a sensitivity agreement test of chronic migraine against others (chronic TTH and other headaches), the proportion of concordance was 0.91, κ = 0.53 (95% CI, 0.28 to 0.79), κ_max_ = 0.81 and PABAK = 0.81 (95% CI, 0.70 to 0.92), very good agreement.

**Table 4:** Frequency of agreement and disagreement between nurse and doctor classification interviews re-classified according to case notes

		Nurses’ classification			
		Chronic migraine	Chronic tension type headache	Others	Total
Doctors’ classification	Chronic migraine	90	1	6	97
	Chronic tension type headache	3	3	0	6
	Others	0	1	3	4
	Total	93	5	9	107

### Nurse feedback

Five nurses completed the short online survey answering questions about their experience of the training and preparation to conduct the classification interviews and their confidence using the logic model. All five nurses felt that the training workshop, one-to-one with a member of the study team and training manual had prepared them adequately to carry out the classification interviews and reported increased confidence the more interviews they completed. They considered the classification guide essential to use when they did the interviews and thought it would be difficult to do the telephone interviews without using the guide. The main challenges described by the nurses were: having to rely on participant recall to ascertain how many days a participant experienced migrainous features to allow distinguish between probable and definite chronic migraine and the number of days abortive medication had been taken to be able to decide if the participant had MOH.

## Discussion

We have developed and validated a telephone classification interview that can be used by a non-headache specialist to classify common chronic headache disorders. Crucially, in the population studied here, there were no disagreements between nurses and doctors on the identification of important headache features needing further medical consideration. Specifically the two people with features of cluster headache were identified by both the nurses and the doctors. That two people with chronic headache identified from searches of GP records had features of undiagnosed cluster headache is perhaps a noteworthy finding. Whilst we cannot draw any statistical inference from this study it is indicative that there is need for general practitioners to consider unrecognised cluster headache in people consulting for headaches. It also provides some empirical justification for the consensus decision to screen for other primary headache types.

Implementation of the nurse classification interviews went well and they were able to use the logic model to inform their decision making, but found the classification guide essential when conducting the telephone interviews. The interviews were, however, challenging because of problems with participant recall of headache frequency and frequency of migrainous symptoms. Inclusion of a headache diary for three months prior to the interview might allow better documentation and might improve precision of the final classification. The use of a diary is recommended in guidelines [12]. This is, however, in the context of a diagnosis in a clinical situation rather than a classification interview for study entry or for epidemiological research. For our current purpose of running a randomised controlled trial in people recruited from general practice registers, in which use of a prospective diary is part of the active intervention, then adding a diary to the pre-entry classification interview is inappropriate. It would be likely to attenuate any therapeutic effect observed in the trial and needlessly prolong the already complicated recruitment process.

The greatest disagreement was between the classifications of probable or definite chronic migraine. The issue here not being the presence or absence of migrainous features but rather the exact frequency. These distinctions are likely to be labile because any delay between interviews (median 32 days) may change final classification, there may be problems with recall bias, and the doctor interviews might have been affected by panel conditioning [13]. Whilst important for describing the population of interest for the CHESS trial in practical clinical terms the distinction between probable or definite chronic migraine may be relatively less important for clinical management. Decision on the choice of migraine prophylaxis and acute treatments are likely to be driven more by the overall clinical picture and patient choice. Thus we are confident that the agreement between nurses and doctors is good enough to inform the selection of people who might benefit from an education and self-management support intervention for chronic headaches which itself includes identifying headache type.

Whilst no substitute for a detailed clinical diagnosis, informed by at least three months of detailed headache diary data we consider our approach is a substantial advance in improving the quality of classification in people living with chronic headaches. Despite comprehensive diagnostic criteria for headache such as The International Classification of Headache Disorders, 3rd edition (beta version) [2], it can be challenging for non-headache experts to diagnose chronic headaches. [4] We present an approach that can easily be operationalised by non-experts and which identifies people whose headache diagnosis need careful consideration by a headache expert. For the purpose of our study we trained nurses to complete the classification interviews by telephone, but envisage that that interview could be delivered by telephone or face-to-face by non- headache expert nurses or doctors to support more accurate diagnosis and management of chronic headache in primary care.

The logic model was developed based on evidence from a systematic review of existing classification tools [8], and the expertise and consensus of academics, health professionals and people living with chronic headache. We used consensus meeting and a nominal group technique methodology to allow all participants to have an equal voice reaching consensus on the essential components of the classification interview, which is a strength of the study. However we acknowledge limitations to the study, we identified participants who consulted general practice and asked GPs to screen for known serious underlying pathology or secondary causes of headache, and therefore the specificity of the logic model is not tested. Our original sample size was 153, but we only completed 107 paired interviews due to time constraints for recruitment to the study. The independent programme steering committee for the study advised to stop participant recruitment as pragmatically the classification was fit for purpose.

## Conclusions

We have developed a new evidence-based telephone classification interview that can be used by a non-headache specialist in primary care to classify common chronic headache disorders: definite chronic migraine, probable chronic migraine and chronic TTH and identify MOH; and to identify those who might need a further diagnostic assessment. Level of agreement between interviews by non-headache specialists and headache specialists was moderate; and very good in a sensitivity test following review of headache characteristics.

We are now using the classification interview in a multi-centre randomised control trial because we are confident that it works well for our purposes.

## Additional files

Additional file 1: Voting results from the consensus conference pleanary session. (DOCX 30 kb)

## Abbreviations

CHESS: Chronic Headache Education and Self-management Study
CSF: Cerebrospinal fluid
GP: General Practitioner
MOH: Medication overuse headache
PABAK: Prevalence-adjusted bias-adjusted kappa
TTH: Tension Type Headache
