# Supplementary material for: Development and validation of a telephone classification interview for common chronic headache disorders
Source: J Headache Pain. 2019 Jan 8;20(1):2. doi: 10.1186/s10194-018-0954-z (PMC6734413; doi:10.1186/s10194-018-0954-z)
Supplement: Supplementary file 1 — Voting results from the consensus conference pleanary session. (DOCX 30 kb) [file 10194_2018_954_MOESM1_ESM.docx]

**Supplementary material**

Table 1: Voting results for the question*: What do we need to know from a person to exclude secondary headaches?*

| **Question** | **Consensus Vote (%)** | **Keep in?** |
| --- | --- | --- |
| For how long have you been having your frequent headaches? | 100 | Yes |
| Age of onset over 50 years of age | 57 | No |
| Have you always been prone to headaches?” | 48 | No |
| Are you unwell? | 27 | No |
| Focal neurological deficits – Aside from the headache is there anything else that isn’t working? | 63 | No |
| How did the headache begin? | 50 | No |
| Have you been to the optician? | 23 | No |
| Medication | 55 | No |
| Do you feel you need a brain scan? | 0 | No |
| Change in character/patterns of headaches? | 70 | No |
| What triggers headache? | 75 | Yes |
| Causal relationship to event/trauma? | 75 | Yes |
| Is headache postural or positional? (high/low pressure headaches) | 88 | Yes |
| Is there impulse/cough/Valsalva | 91 | Yes |
| Pulsatile OR positional tinnitus/visual obscuration (IHH)/visual loss (GCA)? | 79 | Yes |
| Exertion/exercise? | 48 | No |
| Associated symptoms: Sleep apnoea - Sleepy or not during daytime? Lack of sleep refreshment? | 50 | No |
| Speed of onset - Did the headache reach maximum intensity within 5 minutes? | 52 | No |
| How many headache free days do you have in a month? | 67 | No |
| Did the headaches come on soon after starting new medications? | Consensus |  |
| Are you aware of any (secondary) cause for your headache? | 57 | No |
| Do you have any persistent neurological symptoms? Such as change in vision, numbness, weakness, blackouts | 82 | Yes |
| In your past medical history have you had a: persistent infection, clotting tendency, bleeding tendency, known immune deficiency | 52 | No |

Table 2: Voting results for the question*: What do we need to know from a person to exclude primary headaches other than chronic migraine and tension type headache?*

| **Question** | **Consensus Vote (%)** | **Keep in?** |
| --- | --- | --- |
| When did the headache start? | 59 | No |
| How long do your headaches last? | 86 | Yes |
| How did the headache start? | 29 | No |
| Location**:** Is it side-locked?  Is it localised to a specific area? | 90  57 | Yes  No |
| How painful?  Type of pain | 44  Consensus | No |
| Are most of the headache attacks associated with autonomic features? (redness of the eye, watering of the eyes, nasal stuffiness, forehead sweating, eyelid swelling/ puffiness) | 86 | **Yes** |
| Triggers | 48 | No |
| Overall frequency | 86 | Yes |
| Pattern of headache - morning/ evening/time of day**/**time of month**/**time of year  Does your headache only ever wake you in the night? | 95  68 | Yes  No |
| Medication | Consensus | No |
| Family history | Consensus | No |
| Co-morbidities | Consensus | No |
| Neck Pain | Consensus | No |
| Visual disturbance | Consensus | No |
| Do you feel you need a brain scan? | 5 | No |
| New daily persistent headache (Continual headache from onset with no remission) | 84 | Yes |
| Do you experience agitation/restlessness at peak of headache? | 91 | Yes |
| Thinking about your main headache, which description fits best?   - Cardinal symptoms of trigeminal neuralgia - Cardinal symptoms of cluster headache | 55 | No |

Table 3: Voting results for the question: *What do we need to know from a person to distinguish between chronic tension-type headache and chronic migraine?*

| **Question** | **Consensus Vote (%)** | **Keep in?** |
| --- | --- | --- |
| Severity | 61 | No |
| Headache character: Location/ Pulsatile/Non-pulsatile/ Unilateral/Bilateral/Movement sensitivity/Duration of symptoms/ Quality of pain | 83 | Yes |
| Associated symptoms: Nausea/ Vomiting/ Photophobia/ Phonophobia/ Osmophobia/ Dizziness/ Allodynia | 96 | Yes |
| Does it interfere with home or work? | 92 | Yes |
| Headache triggers | 57 | No |
| Aura or not | 74 | Yes |
| Relieving factors “If you could, what would you prefer to do to relieve your headache?” | 52 | No |
| Do you have at least 8 days of headaches with migrainous features per month? | 46 | No |
| Do you have a history of episodic migraines | 70 | No |
| Do you headaches respond to triptans? | 57 | No |
| Does your menstrual cycle affect your headaches? | 67 | No |
| Frequency | 50 | No |
| Migraine features | Consensus | No |
| Behaviours | Consensus | No |
| How severe is your pain when it’s at its worst? | Consensus | No |
| Have you attended or considered attending accident and emergency because of your headache? | 25 | No |
| Is there anything else troubling you? | 4 | No |
| Thinking about your main headache, which description fits best? Cardinal symptoms of trigeminal neuralgia, Cardinal symptoms of cluster headache, Cardinal features of chronic migraine – Lipton, HUNT, Lainez, Cardinal features of tension type headache, None of the above | 39 | No |

Table 4: Voting results for the question: *What do we need to know from a person to identify medication overuse headache?*

| **Question** | **Consensus Vote (%)** | **Keep in?** |
| --- | --- | --- |
| Do you have more days with headache than without? | 73 | No |
| Which medications are you currently taking for headache? | 100 | Yes |
| Over the counter or prescribed medication? | 100 | Yes |
| Have you been taking you (medication) for headache more than 10 days in a month (triptans/ opiates/ mixed) OR 15 days in a month (Paracetamol) + NSAIDS | 95 | Yes |
| How many days per month do you take pain killers (in general – not necessarily for headaches)?  Have you been using pain killers for more than 3 months? | 86  Consensus | Yes  Yes |
| What pain killers do you take? | 95 | Yes |
| Are you receiving more than one type of medication? | 91 | No |
| Are you taking the analgesia daily or only when you have a headache? | 73 | No |
| Why do you take the medication? | 80 | Yes |
| Have you got any other painful conditions? | 77 | Yes |
| Co-morbidities | 42 | No |
| Do you always carry some painkillers with you? | 59 | No |
| Do you keep a headache diary? | 59 | No |
| Are you dependent on your medication to function? | 59 | No |
